# Manual correction of semi-automatic three-dimensional echocardiography is needed for right ventricular assessment in adults; validation with cardiac magnetic resonance

**DOI:** 10.1186/1476-7120-10-1

**Published:** 2012-01-06

**Authors:** Ellen Ostenfeld, Marcus Carlsson, Kambiz Shahgaldi, Anders Roijer, Johan Holm

**Affiliations:** 1 Department of Cardiology, Malmö, Skåne University Hospital, Sweden; 2 Department of Cardiology, Karolinska University Hospital, Huddinge, Stockholm, Sweden; 3 Department of Clinical Physiology, Lund, Skåne University Hospital, Sweden; 4 Department of Cardiology, Lund, Skåne University Hospital, Sweden; Lund University, Sweden

**Keywords:** adult, clinical, three-dimensional echocardiography, magnetic resonance, right ventricle, volumes, function

## Abstract

**Background:**

Three-dimensional echocardiography (3DE) and semi-automatic right ventricular delineation has been proposed as an appropriate method for right ventricle (RV) evaluation. We aimed to examine how manual correction of semi-automatic delineation influences the accuracy of 3DE for RV volumes and function in a clinical adult setting using cardiac magnetic resonance (CMR) as the reference method. We also examined the feasibility of RV visualization with 3DE.

**Methods:**

62 non-selected patients were examined with 3DE (Sonos 7500 and iE33) and with CMR (1.5T). Endocardial RV contours of 3DE-images were semi-automatically assessed and manually corrected in all patients. End-diastolic (EDV), end-systolic (ESV) volumes, stroke volume (SV) and ejection fraction (EF) were computed.

**Results:**

53 patients (85%) had 3DE-images feasible for examination. Correlation coefficients and Bland Altman biases between 3DE with manual correction and CMR were r = 0.78, -22 ± 27 mL for EDV, r = 0.83, -7 ± 16 mL for ESV, r = 0.60, -12 ± 18 mL for SV and r = 0.60, -2 ± 8% for EF (p < 0.001 for all r-values). Without manual correction r-values were 0.77, 0.77, 0.70 and 0.49 for EDV, ESV, SV and EF, respectively (p < 0.001 for all r-values) and biases were larger for EDV, SV and EF (-32 ± 26 mL, -21 ± 15 mL and - 6 ± 9%, p ≤ 0.01 for all) compared to manual correction.

**Conclusion:**

Manual correction of the 3DE semi-automatic RV delineation decreases the bias and is needed for acceptable clinical accuracy. 3DE is highly feasible for visualizing the RV in an adult clinical setting.

## Introduction

Assessment of the right ventricular volumes and function is of great importance in the diagnosis of various heart diseases e.g. pulmonary hypertension and congenital heart disease [1-3], for the choice of therapeutical strategies [4] and not least of prognostic value [5-7].

Two-dimensional echocardiography (2DE) is the most commonly used clinical imaging modality in the evaluation of the right ventricle (RV). The complex geometrical structure of the RV with both a crescent shape and an outspread inflow and outflow tract requires a combination of several different scan planes for estimation of size and function with 2DE. M-Mode and tissue Doppler imaging of the free lateral wall of the RV are measurements in one point and are used as surrogates for the RV function. Hence, current echocardiographic techniques are not suitable for calculating right ventricular volumes and function accurately with a simple algorithm.

Cardiac magnetic resonance imaging (CMR) is currently the gold standard for quantification and monitoring volumes and function of the RV. However CMR is not available at all centres, the equipment is expensive and bedside acquisition is not possible. Some patients cannot undergo CMR because of claustrophobia or implants such as pacemakers and implantable defibrillators (ICD), even if some implanted cardiac devices is becoming a relative contraindication for CMR [8,9].

Three-dimensional echocardiography (3DE) potentially offers a full volume assessment of the RV overcoming the complex geometry problem. Various algorithms have been applied to assess the RV with 3DE, such as multiple planes around the same centreline [10-13], and parallel multislicing [14-17] leaving room for interpolation among planes and slices. Recent studies have used a new tool for RV volume rendering with a semi-automatic dedicated algorithm. The main focus has been patients with congenital heart disease [18-20] and young healthy adult populations [21,22]. Adult patients with acquired heart disease were recently evaluated with the semi-automatic dedicated 3DE algorithm [23,24]. Some investigators have manually corrected the semi-automatic delineation in all patients [23], some not at all [18] and some if considered necessary [24]. Thus, it is not clear if manual correction is needed for clinical use of 3DE.

Therefore, our study was designed to assess how manual correction of the semi-automatic delineation with 3DE influences the accuracy of RV volumetric and functional measurements compared to CMR in a clinical setting with a wide range of adult patients. We also examined the feasibility of visualizing the RV.

## Materials and methods

### Study population and design

62 non-selected patients referred to a clinically indicated transthoracic echocardiography (2DE) or CMR were included. The patients with clinically indicated echocardiography were examined with an additional research related CMR, and patients with clinically indicated CMR were examined with an extra echocardiographic examination. The clinical CMR had complementary indication to a formerly performed echocardiography; e. g. RV volumes and function in suspected arrhythmogenic right ventricular cardiomyopathy, quantification of regurgitations in the aortic or pulmonary valves or definition of infarction size or viability of the myocardium. All patients underwent 2DE in concordance to ASE recommendations [25], three-dimensional echocardiography (3DE) and CMR. 3DE data was recorded directly in continuity to the 2DE examination. Patients were examined with 3DE and CMR with an average of 2 ± 4 days apart and thirty eight patients within the same day. Exclusion criteria was standard contraindication to CMR including claustrophobia (n = 1).

The study was approved by the local ethics committee. All patients gave written informed consent.

### Three-dimensional echocardiography

Data acquisition: 3DE with full volume and harmonic imaging was recorded over 4-7 heart cycles with a matrix array transducer. All recordings were ECG-gated and performed with a breath-hold technique. The first 29 patients were recorded with an x4 transducer and Sonos 7500 (Sonos 7500, Philips Healthcare, Andover, Mass., USA) and the following 33 patients with an x3-1 transducer and iE33 imaging system (Philips Healthcare). The apical view was used for recording, though with a more off-axis approach to be able to include the entire RV. Depth, sector size, angel and focus were adjusted to focus the region of interest to the RV. Two to four recordings were acquired of the RV. The data sets were saved through a digital format to a workstation connected to a TomTec server (TomTec Imaging Systems, Unterschleissheim, Germany) for further analyses.

**Figure 1:**
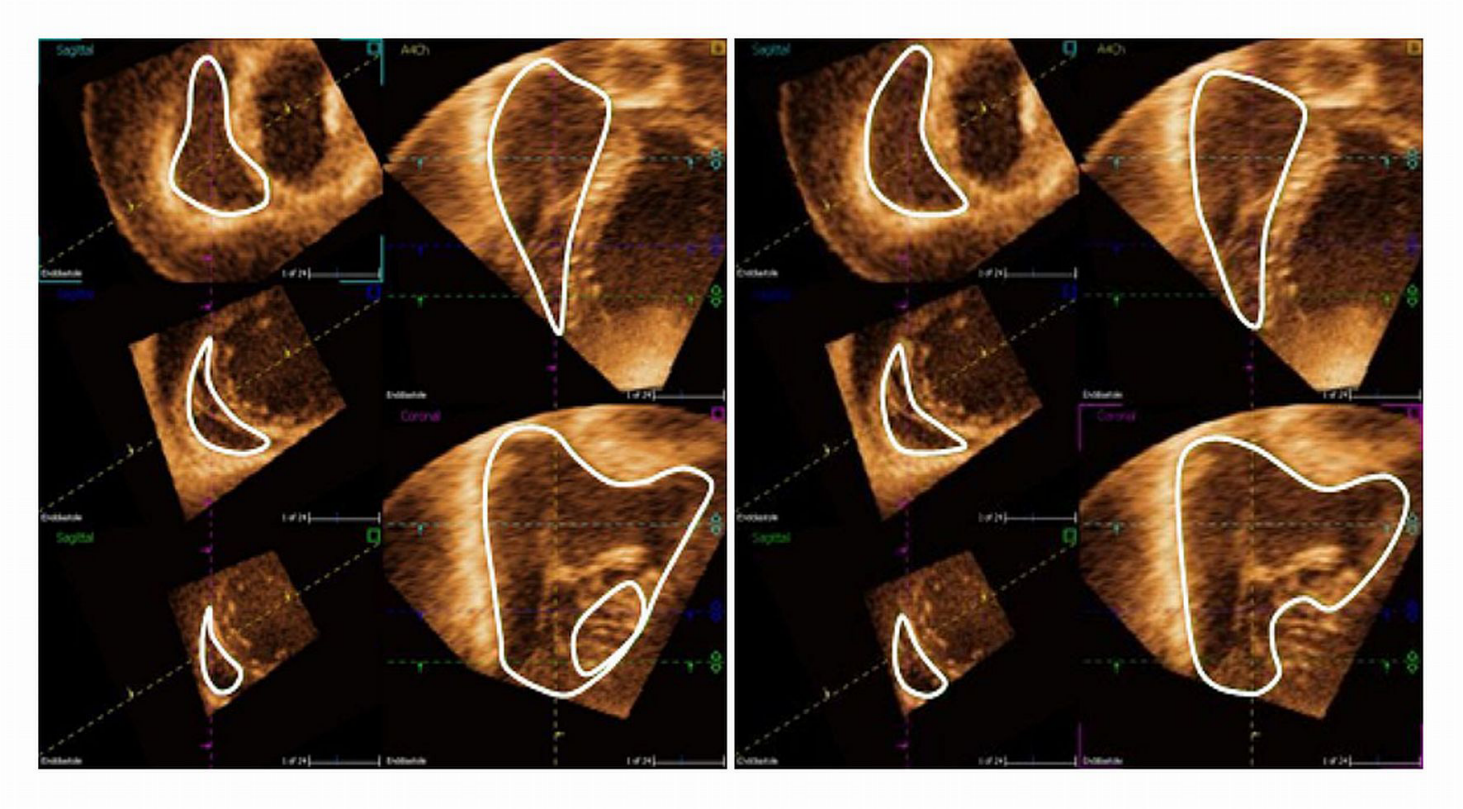
**Semi-automatic tracing of the right ventricle without and with manual correction**. Semi-automatic tracing of the right ventricle in end-diastole with the RV function analysis program showing the endocardial contour detection without (left image) and with (right image) manual correction. In both images: Upper right image is the 4 chamber view; lower right image is the coronal view with the apex downwards, the tricuspid valve to the upper left and the pulmonary valve to the upper right in the image; the three left images are the short-axis views in three different levels; whereas the upper image here is closer to the base and the lower image closer to the apex. The levels of short-axis are changeable and the semi-automatic endocardial rendering is manually corrected in all images and in multiple levels. The uncorrected semi-automatic delineation is less pliable in the anterior and basal part of the RV.

Data analysis: RV data were interpreted with dedicated software 4D RV-Function^© ^(TomTec Imaging Systems). Before tracing, end-systole and end-diastole were defined as the smallest and largest cavity, respectively. The endocardium was traced manually in end-systole and end-diastole in the 4-chamber, short-axis and coronal views. The software algorithm then computed semi-automatic endocardial border detection over the whole heart cycle. Thereafter manual correction was done to optimize the endocardial border delineation in all patients (Figure 1).

The manually corrected and uncorrected (Figure 1) semi-automatic measurements were noted and evaluated for difference in accuracy.

The quality of imaging was judged on a 4-graded scale (1: not visualized, 2: fair, 3: good and 4: excellent) depending on visible endocardium. Acquisitions where one of the four to seven sub volumes were dislocated in the merged full volume data set (also called stitching artefacts) were not used for measurements.

### Cardiac magnetic resonance

#### Data acquisition

A 1.5 T magnetic resonance imaging scanner (Philips Intera, Philips Healthcare, Andover, Mass., USA) with a cardiac synergy coil was used to acquire cine images in the short-axis, long axis 4-chamber and transversal planes during end-expiratory apnoea and ECG-gating. Parallel short-axis images were acquired covering the whole heart from the atria to the apex as well as parallel transversal images covering the whole heart from the diaphragm and up to the great vessels. Typical image parameters were: slice thickness of 8 mm, slice gap of 0 mm, pixel size 1.5 × 1.5 mm, repetition time 2.8 ms, echo time 1.4 ms and flip angle 60°.

#### Data analysis

**Figure 2:**
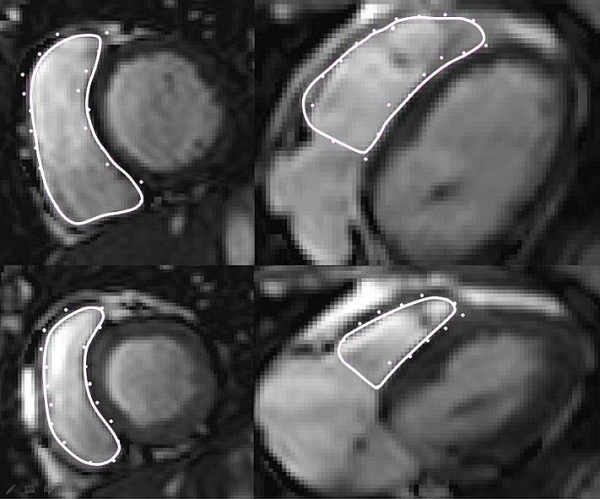
**Cardiac magnetic resonance of the right ventricle**. Manual planimetry of the endocardial contour (full lines) is shown of the right ventricle in cine images. Dots show the traced contours from the corresponding planimetry of short-axis and transversal planes, respectively. The top images are end-diastole and the bottom images are end-systole. To the left are shown the short axis plane and to the right the transversal plane.

All images were evaluated using freely available off-line analysis software (Segment 1.8 R1275, http://segment.heiberg.se) [26]. End-systole and end-diastole were defined as with 3DE. Endocardial contours were traced manually in end-systole and in end-diastole in both transversal and in short axis planes using established methods [27-29]. Planimetry of each of the imaging planes were superimposed to the other (Figure 2), as well as to the long axis 4 chamber to ensure the definition of the RV against encountering structures such as the right atrium, the myocardium, the aorta and the pulmonary artery. The end-systolic (ESV) and end-diastolic (EDV) volumes were computed. The mean values of the two imaging planes (short axis and transversal) were used for comparison with 3DE.

Endocardial tracings of the two imaging modalities were performed blinded to prevent influence on the analyses.

### Statistical Analysis

Data was analysed with Microsoft^® ^Office Excel 2003 and SPSS version 19.0 (SPSS Inc, Chicago, IL, USA). All results are expressed as a mean ± SD. Linear regression analysis with Pearson correlation coefficient and bias according to Bland-Altman [30] were used to compare 3DE and CMR measurements. Student's paired t-test was used to investigate statistical differences between modalities and independent t-test for statistical differences in measurements between grades of image quality. The 2-sided probability value of P < 0.05 was considered to be significant.

## Results

### Patient characteristics

We included 62 patients (22 women, 40 men; ages 55 ± 16). Patient characteristics are presented in table 1. Twenty one patients had impaired right ventricular function in the meaning of ejection fraction below fifty percent. Of those were three under investigation for arrhythmogenic right ventricular cardiomyopathy. The others had impaired right ventricular function due to left-sided diseases, such as mainly congestive heart failure and/or ischemic heart disease (sixteen patients). One patient had hypertrophic cardiomyopathy and one patient had aortopathy with significant aortic regurgitation.

**Table 1:** Patient characteristics

	n = 62	
Age, year	55 ± 16	(18-83)
Heart rate, beats/min	68 ± 13	(48-106)
Body surface area, m^2^	2.0 ± 0.2	(1.3-2.7)
Body mass index, kg/m^2^	26 ± 4	(17-35)
Female	35%	
Sinusrhythm	95%	
Atrial fibrillation/flutter	5%	
Ischemic heart disease	37%	
Congestive heart failure	24%	
Dilated cardiomyopathy	10%	
Atrial fibrillation or flutter	10%	
Aortopathy	6%	
Hypertrophic cardiomyopathy	11%	
Perimyocarditis	5%	
Ventricular arrhytmia	19%	
Sarcoma in the heart	2%	
Valvular heart disease	19%	

Data expressed as mean ± SD (range) or as percentages. Indication for examination is noted. Some patients had more than one indication for examination.

Fifty three of the patients (85%) were feasible for evaluation with 3DE and were used for calculations; of those, 5 had excellent acoustic window, 22 good visualisation, and 26 fair acoustic windows.

### Comparisons with CMR

**Figure 3:**
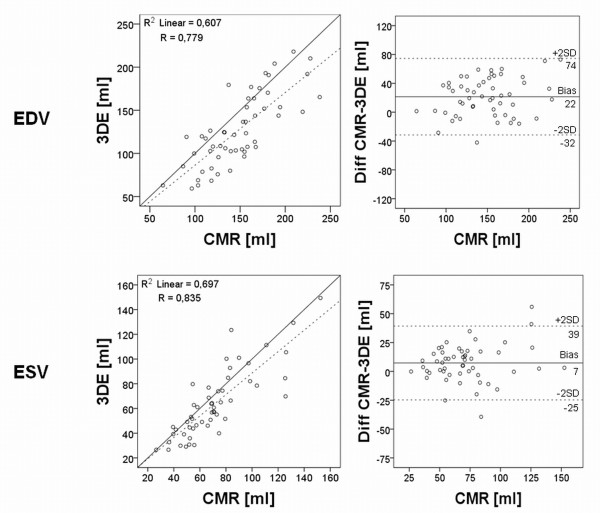
**Regression and Bland-Altman analyses of right ventricular end-diastolic and end-systolic volume with manual correction**. To the left is shown the linear regression analysis of right ventricular end-diastolic volume (EDV) and end-systolic volume (ESV) using 3-dimensional echocardiography (3DE) images against cardiac magnetic resonance (CMR), when manually correcting the semi-automatic delineation. Dashed line shows the linear regression line and full line the identity line. To the right is shown the Bland-Altman plot for EDV and ESV. The full lines show bias and the dashed lines are the limits of agreement (LOA) of ± 1.96 standard deviation (SD).

The correlation coefficient (r) of the manually corrected 3DE compared to CMR was 0.78 for EDV, 0.83 for ESV, 0.60 for stroke volume (SV) and 0.60 for ejection fraction (EF) with p-values < 0.001 for all (Figure 3+4).

**Figure 4:**
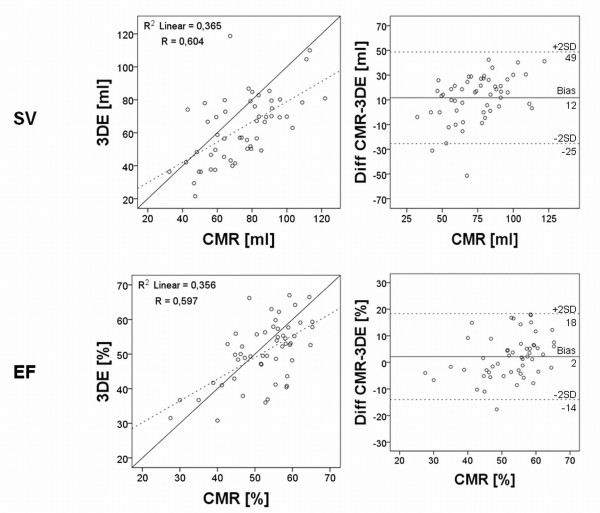
**Regression and Bland-Altman analyses of right ventricular stroke volume and ejection fraction with manual correction**. To the left is shown the linear regression analysis of right ventricular stroke volume (SV) and ejection fraction (EF) using 3-dimensional echocardiography (3DE) images against cardiac magnetic resonance (CMR), when manually correcting the semi-automatic delineation. Dashed line shows the linear regression line and full line the identity line. To the right is shown the Bland-Altman plot for SV and EF. The full lines show bias and the dashed lines are the limits of agreement (LOA) of ± 1.96 standard deviation (SD).

Corrected 3DE consequently underestimated the volumes with mean values of 127 ± 41 ml for EDV, 64 ± 28 ml for ESV and 63 ± 20 ml for SV, with p-values ≤ 0.001 for all (Table 2). EF on the other hand was not significantly different (p = 0.08). Biases was for EDV -22 ± 27 mL (-15 ± 18% of the mean CMR-derived EDV value), for ESV -7 ± 16 mL (-10 ± 21% of the mean ESV value), for SV - 12 ± 18 mL (-15 ± 26% of the mean SV value) and for EF -2 ± 8% (-3 ± 16% of the mean EF value) (Figure 3+4).

**Table 2:** Results of mean values and biases of 3DE and CMR analyses of the right ventricular volumes and function.

		**CMR**	**Manually corrected 3DE analysis**			**Uncorrected semi-automatic 3DE**		
				
	**3DE image quality**	**Mean ± SD**	**Mean ± SD**	**Bias**		**Mean ± SD**	**Bias**	
						
**EDV**	**2+3+4**	148 ± 38 mL	127 ± 41 mL^†^	-22 ± 27 mL	-15 ± 18%	117 ± 38 mL† ‡	-32 ± 26 mL‡	-21 ± 17%
	**3+4**	141 ± 37 mL	124 ± 36 mL†	-18 ± 24 mL	-11 ± 17%	113 ± 35 mL† ‡	-28 ± 25 mL‡	-19 ± 18%
**ESV**	**2+3+4**	71 ± 27 mL	64 ± 28 mL†	-7 ± 16 mL	-10 ± 21%	64 ± 24 mL†	-8 ± 17 mL	-10 ± 23%
	**3+4**	68 ± 28 mL	61 ± 24 mL†	-7 ± 12 mL	-9 ± 18%	63 ± 25 mL	-5 ± 14 mL	-6 ± 22%
**SV**	**2+3+4**	74 ± 20 mL	63 ± 20 mL†	-12 ± 18 mL	-15 ± 26%	55 ± 19 mL† ‡	-21 ± 15 mL‡	-28 ± 18%
	**3+4**	71 ± 20 mL	63 ± 17 mL†	-9 ± 17 mL	-9 ± 26%	50 ± 30 mL† ‡	-21 ± 13 mL‡	-27 ± 15%
**EF**	**2+3+4**	53 ± 9%	51 ± 9%	- 2 ± 8%	-3 ± 16%	46 ± 9%† ‡	-6 ± 9%‡	-11 ± 18%
	**3+4**	52 ± 8%	52 ± 8%	-1 ± 7%	-2 ± 14%	45 ± 8%† ‡	-7 ± 7%‡	-12 ± 14%

Results of mean values and biases of cardiac magnet resonance imaging (CMR), manually corrected semi-automatic and uncorrected semi-automatic three-dimensional echocardiography (3DE) analysis. Means expressed with ± standard deviation (SD). 3DE image quality: 2 means fairly visualized, 3 good and 4 excellent visualized. n = 53 for 2+3+4 and n = 27 for 3+4.

†: compared to CMR, p ≤ 0.001; ‡: compared to corrected 3DE, p ≤ 0.001; No significant differences in means and biases between image quality more than 2 compared to less than 3.

**Figure 5:**
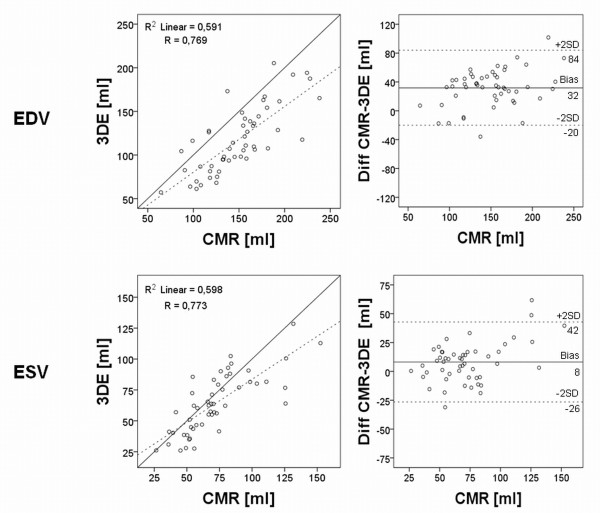
**Regression and Bland-Altman analyses of right ventricular end-diastolic and end-systolic volumes without manual correction**. Results show linear regression analysis and Bland-Altman analysis of right ventricular end-diastolic and end-systolic volumes without manual correction of the semi-automatic volume delineation. Explanations as in figure 3.

The uncorrected semi-automatic 3DE had r-values of 0.77 for EDV, 0.77 for ESV, 0.70 for SV and 0.49 for EF compared to CMR (p < 0.001 for all) (Figure 5+6). The uncorrected analysis underestimated the volumes as well as function with mean values of 117 mL ± 38 mL for EDV, 64 ± 24 mL for ESV, 53 ± 19 mL for SV and 46 ± 9% for EF with p-values ≤ 0.001 for all, when compared to CMR. The biases for uncorrected EDV, SV and EF were larger compared to the manually corrected assessment (EDV -32 ± 26 mL, SV -21 ± 15 mL and EF -6 ± 9%; p ≤ 0.001 for all) and for uncorrected ESV there was no difference in bias (-8 ± 17 mL; p = 0.7) (Table 2).

**Figure 6:**
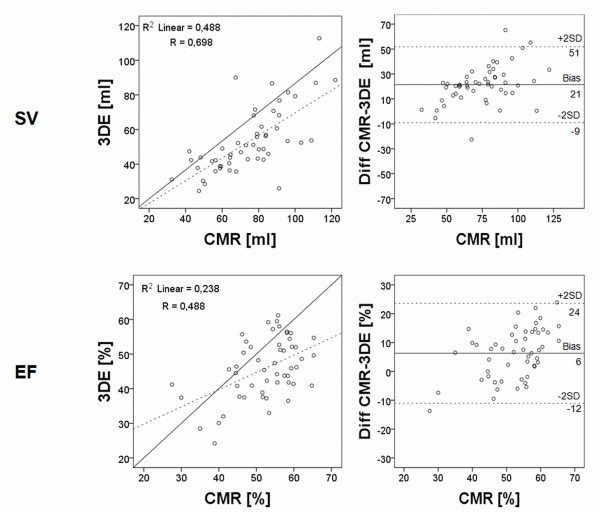
**Regression and Bland-Altman analyses of right ventricular stroke volume and ejection fraction without manual correction**. Results show linear regression analysis and Bland-Altman analysis of right ventricular stroke volume and ejection fraction without manual correction of the semi-automatic delineation. Explanations as in figure 4.

When excluding patients with fair acoustic window (grade 2), there was no change in correlation between corrected 3DE and CMR (EDV, ESV, SV and EF computing r values of 0.78, 0.89, 0.63 and 0.67 with p-values < 0.001 for all), and there were no significant differences in mean values or biases of excellently and well visualized (grade 4 and 3) compared to those fairly visualized (p = 0.34 for EDV, p = 0.99 for ESV, p = 0.09 for SV and p = 0.17 for EF) (table 2). When comparing uncorrected 3DE, without fairly visualized patients, to CMR the r-values were 0.76, 0.84, 0.78 and 0.60 for EDV, ESV, SV and EF, respectively (p < 0.001 for all) and had no difference in means and biases compared to those fairly visualized (p-values of 0.45 for EDV, 0.58 for ESV, 0.43 for SV and 0.72 for EF) (table 2).

All patients were manually corrected in the semi-automatic delineations. The extent of the manual correction, described by the relative difference between the manually corrected and semi-automatic delineation, had a mean of 10 ± 19% for EDV (range -28 to 77%), 0 ± 17% for ESV (range -35 to 40%), 23 ± 38% for SV (range -26 to 206%) and 11 ± 18% for EF (range -24 to 74%). The smallest relative difference was 0% for EDV, 0% for ESV, -1% for SV and 1% for EF.

The manually corrected 3DE analysis was more time consuming 13'37'' ± 4'07'' than uncorrected 3DE analysis 3'44'' ± 1'36'' (p < 0.001) and CMR transversally 11'34'' ± 5'35'' (p < 0.05) but not CMR short-axis 12'11'' ± 5'09'' (p = 0.05).

## Discussion

This study has shown that manual correction of the semi-automated 3DE of the RV is needed as the bias increases without manual correction. We found underestimation of end-diastolic, end-systolic and stroke volume, but not ejection fraction using CMR as a reference.

3DE was feasible for imaging of the RV in a large proportion (85%) of adult patient with unselected pathology.

**Figure 7:**
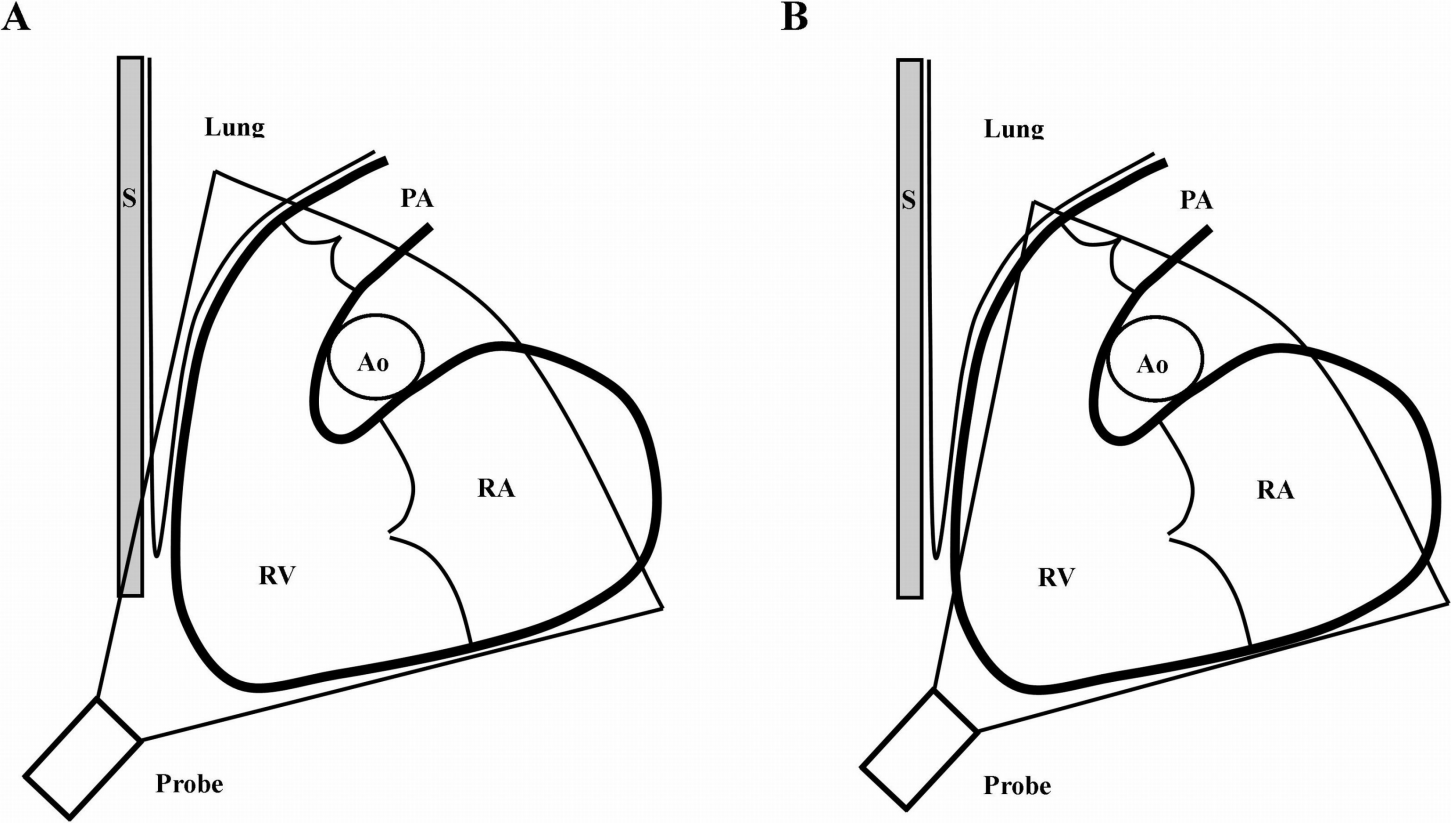
**Image difficulties with 3DE of the right ventricle**. The right ventricle (RV) is depicted with the adjacent structures of the right atrium (RA), the pulmonary trunk (PA), the sternum (S), the ascending aorta (Ao) and lung tissue. A: shows how the sternum or lung tissue can shadow the imaging of the RV especially the anterior part of the right ventricular outflow tract (RVOT). B: shows how the anterior part of the RV might not be included in the whole volume when trying to avoid this shadowing.

The 3DE RV volumes were underestimated compared to CMR in our study. The underestimation of the corrected EDV and SV were balanced and therefore EF does not differentiate between the methods (Table 2). The underestimation of volumes is similar to prior studies using semi-automatic dedicated software [14,18,23]. The underestimation of volumes was mainly caused by insufficient coverage of the right ventricular outflow tract (RVOT) and the anterior wall of the RV on 3DE. This occurred despite the attempt to include and put focus on the outflow tract with a lateralized, tilted and rotated image acquisition and to have the tricuspid, the pulmonary valve and the apex in the same scan plane, (Figure 7). In some patients this lateral approach could not be used because of shadowing from lung tissue or costae and therefore image acquisition was moved medially. With the medial approach the pulmonary valve was not in the same scan plan as the tricuspid valve and the RV apex and this cause uncertainty whether the whole RVOT was included in the acquisition. Furthermore, the medial approach also cause difficulties to visualize the RVOT because of shadowing from the proximity to the sternum [31]. Insufficient visualization of the RVOT is a known problem from an earlier 3DE study by Anwar et al who found inadequate RVOT coverage in 48% of patients although this was the region of interest for assessment [32].

Obesity can cause ultrasonic penetrance problems with substantial amount of tissue in front of the heart and in our patient population several patients were obese (BMI ranged from 17-35). On the other hand the underweighed patients can make it difficult to get sufficient probe-skin contact in between the ribs.

The RV is highly trabeculated and sometimes has a prominent moderator band, crest and papillary muscles which make it more difficult to differ from the correct endocardial border with 3DE. These problems also have to be dealt with when reading CMR images. In addition, the anterior papillary muscle or the moderator band can be mistaken for the anterior wall in the short axis image planes with the semi-automatic 3DE; especially, if a lower image quality of the anterior wall and RVOT is present. This was a frequent problem for the semi-automatic delineation in our study.

Trabeculation and papillary muscles were included in the volumes in our study. Mor-Avi et al showed the spatial resolution influences the detection of trabeculation in the left ventricle and recommended as well to include the trabeculae in the cavity when examining the left ventricle with 3DE [33].

We used disc summation for CMR volume calculations and a RV dedicated algorithm for 3DE volume calculations. Our biases could to some extent be explained by differences in the two methods analysis algorithms and might even be explained in the dissimilarities of the imaging acquisition techniques. These differences have been proposed as a reason to the underestimation of 3DE volumes in earlier studies between CMR and 3DE [13,23]. Differences in volumes and not in function are also seen in other studies of other images modalities of the heart e g gated myocardial perfusion SPECT compared to CMR, suggesting that there are inter-modality differences in the calculation of volumes [34].

### Limitations

Five percent of the patients had atrial fibrillation or atrial flutter. Stitching artefacts can occur when combining four to seven sub volumes with very different RR-intervals, hence end-systole and end-diastole in different time frames. In patients with atrial fibrillation/flutter, four-beat ECG gated acquisition seems to have lower left ventricular volumes and EF than single-beat acquisition [35]. It has been suggested that stitching artefacts could be one explanation. In our material all atrial fibrillation/flutter patients were well regulated and had stable heart frequency and therefore there were no stitching artefacts.

The coronal plane in the semi-automatic software was slightly adjustable and had a large open angle to the 4-chamber long-axis plane. In this "blind" angel was e g the anterior, anterolateral and lateral part of the RV and RVOT. This leaves the area hardest to visualize without a long-axis plane to trace or correct in (Figure 1). To be able to trace in a third long-axis plane intersected in and in between the other two long-axis planes could have facilitated the semi-automatic delineation and might have avoided the more time consuming manual correction in several short-axis levels.

In CMR we used both short-axis and transversal orientations, since there can be difficulties to distinguish the RV boundaries at the level of the tricuspid valve and the outflow tract; especially if there is no perpendicular plane to correct in [15,36]. To minimize this limitation we superimposed the tracing from both orientations to each other with the help of the software as shown in Figure 2. The mean values were used for calculations, since there is a systematic difference in short-axis volumes compared to the volumes of a transversal orientation in the acquisition of images [27].

The 3DE frame rate was in average 20 ± 6 frames per cardiac cycle. If both the volume and the heart rate are low, there is a risk that the true end-diastole and end-systole are missed between frames and that could yield differences in 3DE volumes compared to CMR.

Some of the differences could also be explained by the fact that two different 3DE transducers were used in the echocardiographic examinations. Newer and smaller transducers have higher temporal and spatial resolution and that might improve the visualization of the RV in the future.

Fifteen percent of the patients were not feasible for interpretation and poor acoustic window remains to be a major limitation to 3DE as well as to 2DE. Contrast enhancement has been shown to improve 3DE volumetric quantification of the left ventricle and may also improve RV examination with 3DE [37].

## Conclusion

Manual correction of the 3DE semi-automatic delineation of the RV is needed as the biases increase without manual correction when compared to CMR as a reference. We found underestimation of end-diastolic, end-systolic and stroke volume, but not ejection fraction, for the corrected delineation. 3DE is feasible in an unselected adult patient material for visualizing the right ventricle. 3DE may be a useful clinical tool for RV evaluation in the future with considerable better volume accuracy than 2DE, but still not in parity with CMR standard.

## Abbreviations

2DE: 2-dimensional echocardiography; 3DE: 3-dimensional echocardiography; CMR: Cardiac magnetic resonance; EDV: End-diastolic volume; EF: Ejection Fraction; ESV: End-systolic volume; RV: Right ventricle; RVOT: Right ventricular outflow tract; SV: Stroke volume.
